# Superdense Coding Using Higher Dimensional Embedding

**DOI:** 10.3390/e28040387

**Published:** 2026-04-01

**Authors:** Elio Thadhani, Sharjeel Ahmad, Hussain Ali Razvi, Facundo Martin Lopez, Eric Chitambar

**Affiliations:** 1 Department of Physics, Harvey Mudd College, Claremont, CA 91711, USA; ethadhani@hmc.edu; 2 Department of Physics, University of Illinois Urbana-Champaign, Urbana, IL 61801, USA; ahmad18@illinois.edu; 3 Department of Computer Science, University of Illinois Urbana-Champaign, Urbana, IL 61801, USA; hrazvi2@illinois.edu; 4 Department of Physics and Astronomy, Carleton College, Northfield, MN 55057, USA; lopezf@carleton.edu; 5 Department of Electrical and Computer Engineering, University of Illinois Urbana-Champaign, Urbana, IL 61801, USA

**Keywords:** quantum communication, superdense coding, quantum entanglement, quantum channel, qubit, qudit

## Abstract

Quantum dense coding is a foundational protocol in quantum communication, allowing two classical bits to be transmitted by sending a single qubit when a maximally entangled pair is shared. In this work, we consider Embedded Dense Coding (EDC)—a generalization of deterministic dense coding that embeds one subsystem into a higher-dimensional Hilbert space. To assess the operational advantage of EDC compared to standard dense coding, we consider the probability of transmission error when fixing the rate of entanglement consumed per classical message sent. We first demonstrate that EDC enables a smaller one-shot transmission error compared to standard dense coding when using quantum channels with nonzero rates of dephasing and loss. We then demonstrate that even with noiseless communication channels, EDC leads to smaller overall errors when the sender and receiver have noisy local processors. This advantage is shown through concrete implementations of EDC on IBM’s Heron processor.

## 1. Introduction

Entanglement is a central resource in quantum communication, enabling protocols such as quantum teleportation and quantum key distribution [1,2]. Shared entanglement, through a process called quantum dense coding, allows classical information to be transmitted more efficiently than is possible classically [3]. In its usual form, quantum dense coding allows Alice to transmit $2\log_{2}d$ bits of classical data to Bob using a *d*-dimensional quantum channel combined with $\log_{2}d$ entangled bits (ebits) shared between them. Several generalizations of dense coding have been explored, including protocols with asymmetric dimensions, probabilistic protocols, and alternative resource trade-offs [3,4,5,6,7,8].

From a conceptual point of view, we can describe the resource trade-off of dense coding as [1,9]

(1) logd[qq]+logd[q→q]≥2logd[c→c].

Here, logd[qq] expresses a total of logd ebits, each ebit having the canonical two-qubit form $|\Phi^{+}\rangle =\frac{1}{\sqrt{2}}\left(|00\rangle +|11\rangle \right)$, while logd[q→q] denotes a noiseless quantum channel that can transmit logd qubits, and 2logd[c→c] expresses a noiseless classical channel that can transmit 2logd bits. The inequality means that having the communication resources on the LHS is sufficient for simulating the communication resources on the RHS. We refer to the dense coding protocol described in Equation (1) as Standard Dense Coding (SDC). The relevant feature of SDC for our purposes is that the entangled system on the sender’s side has the same dimension as the input system to the quantum channel.

However, more general dense coding protocols, such as the one proposed in Ref. [4], can achieve the resource trade-off

(2) $$\begin{array}{c} \log d[\mathrm{qq}]+\log D[q\to q]\geq \log dD[c\to c]. \end{array}$$

In this case, the size of the entanglement shared between the sender and receiver has dimension *d* (more precisely, its Schmidt rank is *d*), while the noiseless quantum channel transmits *D*-dimensional quantum states.

This paper considers dense coding scenarios similar to the one captured in Equation (2). Specifically, we are interested in the situation when d<D and exploring what advantages can be attained when the shared entanglement is smaller than the dimension of the connecting quantum channel. From a practical perspective, this is a very natural scenario to consider since storing high-dimensional entanglement over large distances can be very challenging, and thus in the foreseeable future all quantum communication networks will likely satisfy d≪D.

In order to perform dense coding when d<D, the *d*-dimensional entanglement must be embedded into a larger *D*-dimensional system for transmission over the channel. We refer to protocols of this form as Embedded Dense Coding (EDC), and we describe such a protocol in this paper. Unlike previous works that studied higher-dimensional dense coding protocols [4,5], our interest lies in comparing the performance of EDC with SDC.

To make a fair comparison between different dense coding protocols that use the same *D*-dimensional quantum channel, we fix the entanglement-message rate of a given protocol, which is its *rate of entanglement consumed per classical message sent*. Here, we choose to quantify entanglement in terms of its Schmidt rank, which is a bona fide entanglement measure that is suitable for providing a coarse-grained description of entanglement in the one-shot setting [10,11]. Different dense coding protocols with the same entanglement-message rate are on the same level in terms of resource consumption, and thus it is fair to compare them in terms of other communication metrics such as transmission error. SDC and EDC protocols using a *D*-dimensional quantum channel have the same entanglement-message rate:

(3) $$\frac{1}{D}=\frac{D}{D^{2}}=\frac{d}{dD}.$$

When the quantum channel is noiseless, both these protocols transmit classical messages at this rate without any transmission error. However, if we deviate from the ideal scenario and consider a noisy quantum channel, it is not clear whether SDC and EDC have the same performance in terms of decoding error. The fundamental question studied in this paper is whether EDC offers any advantage over SDC in noisy environments by reducing the transmission error at a fixed entanglement-message rate. We find that this is indeed possible when the quantum communication channel or the local encoding/decoding operations are noisy. Hence, in all realistic scenarios, EDC offers a communication advantage over SDC.

In this paper, we present an Embedded Dense Coding protocol which utilizes isometric embedding (Section 4) and verify that it enables the zero-error transmission of messages under noiseless conditions. We then analytically analyze the performance of EDC when the sender Alice communicates to the receiver Bob over a type of noisy quantum channel known as the dephrasure channel (Section 5). The dephrasure channel encompasses errors due to both dephasing and loss, and it provides a realistic model for studying quantum communication effects [12].

**Figure 1:**
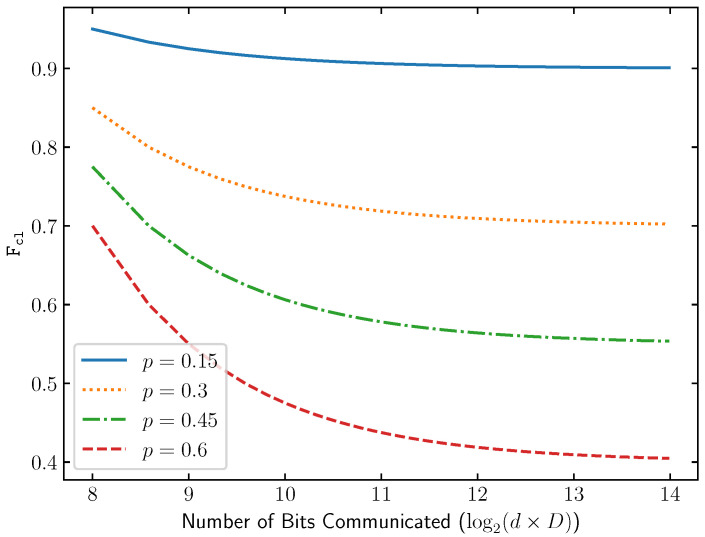
Comparison of the Classical Correlation Fidelity $F_{\mathrm{cl}}$ and the number of bits sent for a given EDC protocol with fixed channel dimension D=128. The dimension of entanglement, *d*, is varied to communicate $\log_{2}\left(d\times D\right)$ classical bits. We show various probabilities of a dephasing *p* and assume an ideal resource as seen in Proposition 1. Here, we can see the trade-off that, using an EDC protocol, one can sacrifice the number of bits transmitted in exchange for a higher one-shot success probability if transmission is subject to dephasing noise.

**Figure 2:**
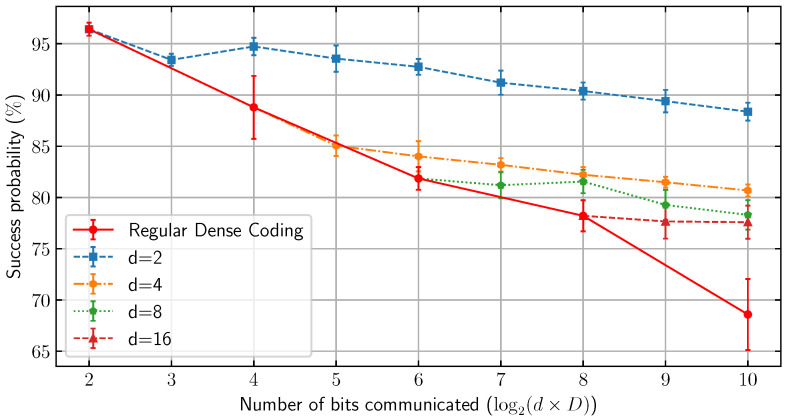
Success probabilities for EDC schemes with varying dimensions implemented on IMB’s Heron processor through ${\mathrm{IBM}}_{\mathrm{Torino}}$. Due to hardware limitations, logical qudits are encoded as physical qubits. We plot the success probability of a given EDC scheme that can communicate a set number of classical bits utilizing an entanglement resource $\rho \in B(H_{B}⊗H_{A}):|A|=|B|=d$. The embedding dimension *D* is varied to compare success probability based on the number of bits communicated and the starting resource. Each plotted branch represents a different starting resource dimension *d*, while the conventional dense coding branch utilizes d=D. For example, 6 bits can be communicated utilizing {d=8,D=8},{d=4,D=16}, and {d=2,D=32}. Each data point was collected over six independent trials, each consisting of 10,000 shots.

Our main findings are as follows. We first observe that the tolerance to dephasing error increases as the embedding dimension *D* increases. Moreover, at a fixed entanglement-message rate $\frac{1}{D}$, EDC always obtains a strictly larger transmission success probability in the presence of dephasing compared to SDC (see Proposition 1). This makes EDC a versatile protocol for one-shot entanglement-assisted classical communication, as it allows for sacrificing the number of bits transmitted in exchange for a higher one-shot success probability when the transmission is subject to dephasing noise (see Figure 1). We then experimentally analyze the performance of EDC when Alice and Bob have noisy local encoding/decoding and their shared entanglement is stored in noisy quantum memory (Section 6). This is done by implementing EDC on IBM’s Heron Processor. By providing an explicit construction, we show that EDC can be realized using a fewer number of encoding/decoding gates, which ultimately leads to a smaller transmission error when compared to conventional dense coding (see Figure 2).

Throughout this work, quantum systems will be denoted by A,B,C, etc., and classical systems will be denoted by X,Y,Z, etc. The Hilbert space associated with a bipartite system AB will be defined as $H_{AB}≔H_{B}⊗H_{A}$ [13]. For a quantum system *A* with associated Hilbert space $H_{A}$, we denote its dimension by $|A|≔\dim H_{A}$. Systems whose representation differs only by primes have equal dimension, i.e., $\left|A|=|A^{\prime }|=|A^{\prime \prime }\right|$. Systems whose representation differs by a subscript, i.e., *A* and $A_{e}$, do not necessarily have the same dimension and are used to differentiate the same system after some projection or embedding process. The space of operators acting on $H_{A}$ is defined as $B(H_{A})$. A pure state ${|\psi \rangle }_{A}\in H_{A}$ has associated density operator $\psi_{A}=|\psi \rangle {\langle \psi |}_{A}\in B\left(H_{A}\right)$. The identity operator acting on $H_{A}$ is denoted $I_{A}$, and the identity map on $B(H_{A})$ is denoted ${\mathrm{id}}_{A}$. Mathematically, every quantum channel $N_{A\to B}:B\left(H_{A}\right)\to B\left(H_{B}\right)$ is described by a completely positive trace-preserving (CPTP) map.

## 2. Background

The generalized Pauli operators provide a useful set of orthogonal operators that act on qudit (*d*-dimensional) quantum state space. These operators play a significant role in the creation of generalized communication protocols.

**Definition** **1** (Pauli Operators [14])**.** *Let the Generalized Pauli X and Z operators acting on a d-dimensional system $H_{B}$ be defined as $X_{d}^{m}$ and $Z_{d}^{n}$ where*

(4) $$X_{d}^{m}=\sum_{j=0}^{d-1}|j⊕m\rangle \langle j|\;\mathrm{for}\;m=0,\ldots ,d-1$$

*and*

(5) $$Z_{d}^{n}=\sum_{k=0}^{d-1}\omega^{kn}|k\rangle \langle k|\;:\omega =e^{i\frac{2\pi }{d}}\;\mathrm{for}\;m=0,\ldots ,d-1.$$

### 2.1. Entanglement

Quantum entanglement has become a fundamental aspect of quantum information theory. References [15,16,17,18] help define different aspects of quantum entanglement, including discussions of maximally entangled pure states and entangled mixed states. In general, a bipartite state $\rho_{AB}$ is considered to be untangled if and only if it can be expressed as a convex combination of product states [19]. Throughout this text, we will focus primarily on utilizing maximally entangled pure states (MES) as communication resources. For *d*-dimensional systems *C* and $C^{\prime }$, a state ${|\Phi \rangle }_{CC^{\prime }}\in H_{C}⊗H_{C^{\prime }}$ is called maximally entangled if it has the form

(6) $${|\Phi \rangle }_{CC^{\prime }}=U_{C}⊗I\;\frac{1}{\sqrt{d}}\sum_{k=0}^{d-1}{|k\rangle }_{C}⊗{|k\rangle }_{C^{\prime }}$$

where $U_{C}$ is an arbitrary unitary operator. The dimension *d* is also called the Schmidt rank of state |Φ〉. A canonical maximally entangled state is given by the choice $U_{C}=I$, which we denote ${|\Phi^{+}\rangle }_{CC^{\prime }}$.

As discussed by [18], for some bipartite state $\rho_{AB}$, we can define the Fully Entangled Fraction. The Fully Entangled Fraction (FEF) is notably not a valid entanglement measure but rather a lower bound on the amount of entanglement present for a given resource [20]. However, the FEF is still a meaningful quantity in the study of quantum communication protocols and is shown to provide useful information when evaluating entanglement applications [21].

**Definition** **2** (FEF [18])**.** *The Fully Entangled Fraction of some state $\rho \in B(H_{C}⊗H_{C^{\prime }})$ is given by*

(7) $$F_{\left|C\right|}\left(\rho \right)=\max_{\Phi }\langle \Phi |\rho |\Phi \rangle$$

*where the maximum is over all |C|-dimension maximally entangled states ${|\Phi \rangle }_{CC^{\prime }}\in H_{C}⊗H_{C^{\prime }}$.*

### 2.2. Dense Coding

Standard dense coding (SDC), originally proposed by [3], enables Alice to communicate $\log_{2}\left(d^{2}\right)$ classical bits by sending Bob one-half of a *d*-dimensional maximally entangled state over a noiseless quantum channel. In the ideal case, Alice and Bob have the maximally entangled state as a shared resource in advance. However, the notion of dense coding can be defined more generally for an arbitrary bipartite state $\rho_{AB}$ and quantum channel $N_{A_{e}\to B_{e}}$. For completion, we state the definition here.

**Definition** **3.** *An N message dense coding protocol from Alice to Bob over channel $N_{A_{e}\to B_{e}}$ is defined in terms of a tuple $D=(\rho_{AB},{\left\{E^{i}\right\}}_{i=0}^{N-1},{\left\{\Pi^{j}\right\}}_{j=0}^{N-1})$, where $\rho_{AB}$ is a quantum state shared between Alice and Bob, ${\left\{E^{i}\right\}}_{i=0}^{N-1}$ is a family of encoding channels with $E^{i}:A\to A_{e}$, and ${\left\{\Pi^{j}\right\}}_{j=0}^{N-1}$ is a POVM acting on systems $BB_{e}$. The protocol goes as follows:*

1. *Alice and Bob each hold their respective subsystems of the bipartite entangled state $\rho_{AB}$, and Alice has a classical message i∈[N]:={0,…,N−1} that she wishes to communicate to Bob.*
2. *Alice encodes the message i into her part of the entangled state $\rho_{AB}$ using the encoding operator $E^{i}:A\to A_{e}$.*
3. *Alice sends Bob subsystem $A_{e}$ over channel $N_{A_{e}\to B_{e}}$.*
4. *Bob measures the joint system $BB_{e}$ using the POVM ${\left\{\Pi^{\mu_{j}}\right\}}_{j=0}^{N-1}$ and guesses that Alice sent message j whenever he obtains measurement outcome j.*

*Overall, this generates a classical channel W:[N]→[N] with transition probabilities given by*

(8) $$W\left(j|i\right)=\mathrm{Tr}[\Pi^{j}\cdot N\circ E^{i}⊗{\mathrm{id}}_{B}\left(\rho_{AB}\right)].$$

When the dimension of the channel input, $D=|A_{e}|$, is larger than the dimension of the system storing Alice’s entanglement, d=|A|, the encoders $E^{i}$ are embedding maps. In this work, we will be exclusively interested in isometric embeddings. These are easily constructed as follows. Let ${\left\{|\theta_{i}\rangle \right\}}_{i=0}^{d-1}$ and ${\left\{|\phi_{i}\rangle \right\}}_{i=0}^{D-1}$ both be an orthonormal basis for systems *A* and $A_{e}$, respectively, with d≤D. Then, the isometric embedding operator $V_{A\to A_{e}}:H_{A}\mapsto H_{A_{e}}$ can be constructed as

(9) $$V_{A\to A_{e}}=\sum_{i=0}^{d-1}|\phi_{i}\rangle \langle \theta_{i}|.$$

For convenience, we denote the action of the isometry on density matrices as the CPTP map $V\left(\rho_{AB}\right)=V\left(\rho_{AB}\right)V^{†}$.

## 3. Performance Criteria

In this work, we evaluate the performance of a dense coding protocol in terms of its average success probability in transmitting classical messages from Alice to Bob, assuming a uniform prior distribution. We refer to this as the Classical Correlation Fidelity, $F_{\mathrm{cl}}$.

**Definition** **4** (Classical Correlation Fidelity [13])**.** *For a classical channel W:[N]→[N] with transition probabilities W(j|i), its Classical Correlation Fidelity $F\left(W\right)\equiv F_{\mathrm{cl}}$ is defined as*

(10) $$F_{\mathrm{cl}}=\frac{1}{N}\sum_{i=0}^{N-1}W\left(i|i\right).$$

*For a dense coding protocol D defined in Definition 3, its Classical Correlation Fidelity is given by*

(11) $$\begin{array}{c} F_{\mathrm{cl}}=\frac{1}{N}\sum_{i=0}^{N-1}\mathrm{Tr}[\Pi^{i}\cdot N\circ E^{i}⊗{\mathrm{id}}_{B}\left(\rho_{AB}\right)]. \end{array}$$

We stress that the choice of performance metric chosen here is best suited for the one-shot setting. This refers to the fact that we are considering dense coding protocols that encode/decode over a single use of both the resource state $\rho_{AB}$ and the channel $N_{A_{e}\to B_{e}}$. In contrast, one could consider applying block-encoding across multiple uses of the resource state or the quantum channel, which is a more familiar information-theoretic problem. When performing asymptotic block encoding, the more relevant figure of merit for classical communication is the Holevo information [22] or the quantum mutual information [23] for entanglement-assisted channel coding.

For example, prior work on Hiroshima has identified dense coding protocols that maximize the Holevo information for a given resource state $\rho_{AB}$ [8]. The construction presented in that work involves a group-covariant unitary encoding. Rather remarkably, this encoding/decoding strategy is optimal for every state $\rho_{AB}$ with fixed dimensions |A| and |B|. However, the construction will not necessarily be optimal when considering the Classical Correlation Fidelity $F_{\mathrm{cl}}$ as a figure of merit, and, in general, optimizing $F_{\mathrm{cl}}$ requires solving a bilinear optimization problem (see Ref. [13] for details). Nevertheless, the EDC protocol we propose in the next section has the same symmetric form as Hiroshima’s, and it turns out to indeed optimize $F_{\mathrm{cl}}$ for the special case of sending half a maximally entangled state over the dephrasure channel.

## 4. Embedded Dense Coding

We now describe an Embedded Dense Coding (EDC) protocol from Alice to Bob, which is defined for a shared resource state $\rho_{AB}$ and any channel $N_{A_{e}\to B_{e}}$ with |A|=|B|=d and $|A_{e}|=|B_{e}|=D>d$. In this protocol, Alice attempts to send Bob one of d·D possible messages μ∈{(k,j):k∈[d],j∈[D]}. The mapping of system *A* into $A_{e}$ is done via an isometric embedding.

Alice first performs the generalized Pauli $Z_{A}^{k}$ operation on her subsystem, encoding the *k*-dit element of his message. As a result, the joint bipartite system is now

(12) $$\rho_{k}=\left(I⊗Z_{A}^{k}\right)\rho \left(I⊗Z_{A}^{k^{†}}\right).$$

She then embeds her subsystem into a higher-dimensional space $H_{A_{e}}$ using an isometric embedding V, as described in Equation (9). We write ϱ:=V(ρ) to denote the embedded form of any density matrix ρ. However, note that it is equivalent for Alice to encode *k after* the embedding process using the operator

(13) $${\hat{Z}}_{A_{e}}^{k}=V_{A\to A_{e}}Z_{A}^{k}V_{A\to A_{e}}^{†}.$$

That is, $\varrho_{k}={\hat{Z}}^{k}\varrho {\hat{Z}}^{k†}$. We will utilize this alternative convention for notational simplicity.

Alice next encodes the index *j* of her message by performing a generalized Pauli $X_{A_{e}}^{j}$ operation on her subsystem. The resulting joint state is now

(14) $$\begin{array}{cc} \varrho^{\mu }:= & \left(I⊗X_{A_{e}}^{j}{\hat{Z}}_{A_{e}}^{k}\right)\varrho \left(I⊗{\hat{Z}}_{A_{e}}^{k^{†}}X_{A_{e}}^{j^{†}}\right). \end{array}$$

In total, the encoder for message μ=(k,j) is given by the CPTP map $E^{\mu }:A\to A_{e}$ given by

(15) $$E^{\mu }\left(\cdot \right)=X_{A_{e}}^{j}{\hat{Z}}_{A_{e}}^{k}\left(\cdot \right){\left(X_{A_{e}}^{j}{\hat{Z}}_{A_{e}}^{k}\right)}^{†}.$$

Note that this encoder is an isometry and the set of encoding operators $X_{B_{e}}^{j}{\hat{Z}}_{B_{e}}^{k}$ are mutually orthogonal. This is consistent with the encoding strategy that maximizes the Holeveo information [8].

The orthogonality of the $X_{A_{e}}^{j}{\hat{Z}}_{A_{e}}^{k}$ naturally suggests the following strategy for decoding. First, let us denote the encoded states as

(16) $$\varrho^{\mu }=\mathrm{id}⊗E^{\mu }\circ V\left(\rho \right).$$

Notice that when choosing ρ=|Ψ〉〈Ψ| to be maximally entangled with Schmidt rank *d*, the encoded states $\varrho^{\mu }$ form an orthonormal set. We can therefore define a decoding POVM for Bob by

(17) $$\Pi_{BB_{e}}^{\mu }=\mathrm{id}⊗E^{\mu }\circ V\left(|\Psi \rangle \langle \Psi |\right),$$

plus an additional effect $I-\sum_{\mu }\Pi_{BB_{e}}^{\mu }$, which projects onto the space orthogonal to all the encoded states. Observe that this construction leads to an error-free transmission of all d·D messages when all resources and communication are noiseless since

(18) $$\mathrm{tr}\left[\Pi_{BB_{e}}^{\mu }\varrho^{\mu }\right]=\mathrm{tr}\left[\Pi_{BB_{e}}^{\mu }\mathrm{id}⊗E^{\mu }\circ V\left(|\Psi \rangle \langle \Psi |\right)\right]=\mathrm{tr}\left[|\Psi \rangle \langle \Psi |\right]=1,\;\forall \mu .$$

While the EDC protocol here is clearly optimal when using a noiseless quantum channel ${\mathrm{id}}_{A_{e}\to B_{e}}$, it is also shown in Appendix A to be optimal for certain types of noisy channels and resources.

**Remark** **1.** *There is freedom in the choice of maximally entangled state |Ψ〉 when defining the decoder in an EDC protocol. For communication over noisy channels, this choice will affect the Classical Correlation Fidelity $F_{\mathrm{cl}}$. Hence, in general, one will need to optimize over the choice of |Ψ〉 to maximize $F_{\mathrm{cl}}$.*

For a general bipartite state $\rho_{AB}$ and a noiseless quantum channel ${\mathrm{id}}_{A_{e}\to B_{e}}$, the Classical Correlation Fidelity of EDC can be expressed in terms of the singlet fraction.

**Lemma** **1.** *EDC over a D-dimensional noiseless quantum channel using bipartite state $\rho_{AB}$ with d=|A|=|B| satisfies $F_{\mathrm{cl}}=F_{d}\left(\rho \right)$.*

**Proof.**  Let |Ψ〉 be the *d*-dimensional maximally entangled state such that $F_{d}\left(\rho \right)=\langle \Psi |\rho |\Psi \rangle$. We use this state to define the decoding POVM in EDC, as in Equation (17). Then for every message μ, we have

(19) $$\begin{array}{cc} p(\mu |\mu ) & =\mathrm{Tr}[\Pi^{\mu }\varrho^{\mu }]\\ \; & =\mathrm{Tr}[E^{\mu }\circ V\left(|\Psi \rangle \langle \Psi |\right)E^{\mu }\circ V\left(\rho \right)]\\ \; & =\langle \Psi |\rho |\Psi \rangle =F_{d}\left(\rho \right), \end{array}$$

where we have used the fact that the EDC encoder and decoder are isometries, and they therefore preserve inner products. Hence, by the definition of the Classical Correlation Fidelity $F_{\mathrm{cl}}$, we have $F_{\mathrm{cl}}=F_{d}\left(\rho \right)$. □

## 5. Noisy Transmission: Dephrasure Quantum Channel

Having established that the EDC protocol enables perfect transmission of d·D classical messages in the case of noiseless communication resources, we now turn to the question whether EDC is robust to noise. We are specifically interested in comparing the performance of EDC to standard dense coding (SDC). Suppose Alice can send Bob quantum information over a *D*-dimensional quantum channel. Recall that SDC involves using an entangled system that has the same dimension as the quantum channel connecting Alice to Bob. We are interested in understanding whether or not there is any advantage in using entanglement and channels with mismatched dimensions, thereby leveraging an EDC protocol. This is a highly practical question since storing high-dimensional entanglement for use in distributed communication protocols is experimentally demanding. We demonstrate that indeed an advantage in using EDC can be found when communication is subject to dephasing and erasure.

More precisely, we consider that Alice and Bob are connected by a dephrasure channel [12], which is a two-parameter CPTP map transforming $A_{e}\to B_{e}$ and having the form

(20) $$\begin{array}{c} N_{p,q}\left(\rho \right)=\left(1-q\right)\left(\left(1-p\right)\rho +\frac{p}{D}\sum_{\ell =0}^{D-1}Z^{\ell }\rho Z^{\ell^{†}}\right)+q\mathrm{Tr}\left(\rho \right)|e\rangle \langle e|. \end{array}$$

with $D=|A_{e}|$. Here, |e〉 is a vector orthogonal to the support of ρ, serving as an erasure flag that the receiver can detect. The parameter q∈[0,1] represents the probability of erasure, while p∈[0,1] represents the probability of completely dephasing the state.

Standard dense coding over a *D*-dimensional dephrasure channel is performed on a D×D state $\rho_{AB}^{\left(D\right)}$ and attempts to transmit $D^{2}$ messages. EDC over the same channel uses a d×d state $\rho_{AB}^{\left(d\right)}$ with d<D and sends a correspondingly smaller number of d·D messages. Note that in both these scenarios, SDC and EDC have the same entanglement-message rate of $\frac{1}{D}$. They are thus comparable in terms of resource consumption. Nevertheless, as one of our main findings, we observe that they differ in their average transmission error.

**Proposition** **1.** *For a D-dimensional dephrasure channel $N_{p,q}$, SDC and EDC protocols using arbitrary entangled resources $\rho^{\left(D\right)}$ and $\rho^{\left(d\right)}$, respectively, have their Classical Correlation Fidelities upper bounded as*

(21) $$\begin{array}{cc} {F_{\mathrm{cl}}}^{\left(\mathrm{SDC}\right)} & \leq \left(1-q\right)\left(\left(1-p\right)F_{D}\left(\rho^{\left(D\right)}\right)+\frac{p}{D}\right)+\frac{q}{D^{2}} \end{array}$$

(22) $$\begin{array}{cc} {F_{\mathrm{cl}}}^{\left(\mathrm{EDC}\right)} & \leq \left(1-q\right)\left(\left(1-p\right)F_{d}\left(\rho^{\left(d\right)}\right)+\frac{p}{d}\right)+\frac{q}{dD}. \end{array}$$

*Moreover, these upper bounds can be made tight by using a maximally entangled resource state. In this case, EDC has a greater $F_{\mathrm{cl}}$ whenever p≠0.*

**Proof of** **Proposition 1.** We prove the upper bound of Equation (22); the argument for Equation (21) follows analogously. As in the proof for Lemma 1, let |Ψ〉 be such that $F_{d}\left(\rho \right)=\langle \Psi |\rho |\Psi \rangle$. We use this state to build the decoder of an EDC protocol with the decoding POVM given by Equation (17).

Starting with the state $\rho^{\left(d\right)}$, let $\varrho^{\mu }$ denote the state after Alice applies encoder $E^{\mu }\circ V$ for message μ. System $A_{e}$ is then subject to the dephrasure channel $N_{p,q}$, yielding the transformed state ${\hat{\varrho }}^{\mu }$. Thus, the final state held by Bob prior to the decoding measurement is

(23) $$\begin{array}{cc} {\hat{\varrho }}^{\mu }= & \left({\mathrm{id}}_{B}⊗N_{p,q}\right)\left(\varrho^{\mu }\right)\\ = & \left(1-q\right)(\left(1-p\right)\left(\mathrm{id}⊗E^{\mu }\right)\left(\varrho \right)\\ & +\frac{p}{D}\sum_{\ell =0}^{D-1}\left(I⊗Z_{A_{e}}^{\ell }\right)\left(\mathrm{id}⊗E^{\mu }\right)\left(\varrho \right)\left(I⊗Z_{A_{e}}^{\ell^{†}}\right))+q\rho_{B}⊗|e\rangle \langle e|, \end{array}$$

where $\rho_{B}={\mathrm{tr}}_{A}\left[\rho^{\left(d\right)}\right]$.

**Lemma** **2.** *The encoding operator $E^{\mu }\left(\cdot \right)=X_{A_{e}}^{j}{\hat{Z}}_{A_{e}}^{k}\left(\cdot \right){\left(X_{A_{e}}^{j}{\hat{Z}}_{A_{e}}^{k}\right)}^{†}$ defined in Equation (15) commutes under conjugation with any generalized canonical Pauli $Z_{A_{e}}$ operator as defined in Definition 1.*

**Proof.**  Let $E^{\mu }\left(\cdot \right)=X_{A_{e}}^{j}{\hat{Z}}_{A_{e}}^{k}\left(\cdot \right){\left(X_{A_{e}}^{j}{\hat{Z}}_{A_{e}}^{k}\right)}^{†}$ and $Z_{A_{e}}^{\ell }$ be some generalized pauli operator such that $Z_{A_{e}}^{\ell }\in B\left(A_{e}\right)$. Then,

(24) $$Z_{A_{e}}^{\ell }E^{\mu }\left(\cdot \right){Z_{A_{e}}^{\ell }}^{†}=Z_{A_{e}}^{\ell }X_{A_{e}}^{j}{\hat{Z}}_{A_{e}}^{k}\left(\cdot \right){\left(X_{A_{e}}^{j}{\hat{Z}}_{A_{e}}^{k}\right)}^{†}{Z_{A_{e}}^{\ell }}^{†}.$$

Note, by the Weyl Commutation relation, $Z_{A_{e}}^{\ell }X_{A_{e}}^{j}=\omega Z_{A_{e}}^{\ell }X_{A_{e}}^{j}$ for some ω∈C where |ω|=1. Additionally note that $[Z_{A_{e}}^{\ell },{\hat{Z}}_{A_{e}}^{k}]=0$ since both $Z_{A_{e}}^{\ell }$ and ${\hat{Z}}_{A_{e}}^{k}$ are diagonal. It therefore follows that

(25) $$\begin{array}{cc} Z_{A_{e}}^{\ell }E^{\mu }\left(\cdot \right){Z_{A_{e}}^{\ell }}^{†}= & Z_{A_{e}}^{\ell }X_{A_{e}}^{j}{\hat{Z}}_{A_{e}}^{k}\left(\cdot \right){\left(X_{A_{e}}^{j}{\hat{Z}}_{A_{e}}^{k}\right)}^{†}{Z_{A_{e}}^{\ell }}^{†}\\ = & X_{A_{e}}^{j}{\hat{Z}}_{A_{e}}^{k}Z_{A_{e}}^{\ell }\left(\cdot \right){\left(X_{A_{e}}^{j}{\hat{Z}}_{A_{e}}^{k}Z_{A_{e}}^{\ell }\right)}^{†}=E^{\mu }\left(Z_{A_{e}}^{\ell }\left(\cdot \right){Z_{A_{e}}^{\ell }}^{†}\right). \end{array}$$

□

By Lemma 2, we know that the encoding operator $E^{\mu }$ commutes with the Pauli *Z* twirling channel; thus

(26) $$\begin{array}{cc} {\hat{\varrho }}^{\mu }= & \left({\mathrm{id}}_{A}⊗N_{p,q}\right)\left(\varrho^{\mu }\right)\\ = & \left(1-q\right)\left(\left(1-p\right)\left(\mathrm{id}⊗E^{\mu }\right)\varrho +\frac{p}{D}\left(\mathrm{id}⊗E^{\mu }\right)\left(\sum_{\ell =0}^{D-1}\left(I⊗Z_{A_{e}}^{\ell }\right)\varrho \left(I⊗Z_{A_{e}}^{\ell^{†}}\right)\right)\right)\\ & +q\rho_{B}⊗|e\rangle \langle e|. \end{array}$$

The general effect of Pauli Z twirling on an arbitrary bipartite state $\rho_{BA_{e}}$ is given by

(27) $$\rho_{BA_{e}}\mapsto \frac{1}{|A_{e}|}\sum_{\ell =0}^{|A_{e}|-1}\left(I⊗Z^{\ell }\right)\rho_{BA_{e}}\left(I⊗Z^{\ell^{†}}\right)=\sum_{\kappa =0}^{|A_{e}|-1}\rho_{B}^{\kappa }⊗|\kappa \rangle {\langle \kappa |}_{A_{e}}$$

where $\rho_{B}^{\kappa }:={\mathrm{Tr}}_{A_{e}}\left[\left(I_{B}⊗|\kappa \rangle \langle \kappa |\right)\varrho_{BA_{e}}\right]$. Substituting this into Equation (26) yields

(28) $$\begin{array}{c} {\hat{\varrho }}^{\mu }=\left(1-q\right)\left(\mathrm{id}⊗E^{\mu }\right)\left(\left(1-p\right)\varrho +\frac{p}{D}\left(\sum_{\kappa =0}^{D-1}\rho_{B}^{\kappa }⊗|\kappa \rangle {\langle \kappa |}_{A_{e}}\right)\right)+q\rho_{B}⊗|e\rangle \langle e|. \end{array}$$

We can now calculate the Classical Correlation Fidelity, which we recall is defined as

(29) $$F_{\mathrm{cl}}=\frac{1}{dD}\sum_{\mu =0}^{dD-1}p\left(\mu |\mu \right).$$

By the symmetry of states and assuming equal priors, we have that $F_{\mathrm{cl}}=p\left(\mu |\mu \right)$ for all μ. Furthermore, note that our encoding operators preserve the inner product on the pre-image of our embedding, and, in the case of an erasure flag, |e〉〈e|, we will assume the message is randomly guessed with success probability 1/dD. Thus,

Fcl(EDC)=p(μ|μ)=Tr[ϱ^μΠBBeμ](30)=(1−q)(1−p)Fd(ρ)+(1−q)pTr(∑κ=0D−1ρBκ⊗|κ〉〈κ|Ae)V(|Ψ〉〈Ψ|)+qdD(31)≤(1−q)(1−p)Fd(ρ)+1d(1−q)p+qdD,

where the upper bound follows from the fact that

(32) $$\begin{array}{c} \sum_{\kappa =0}^{D-1}\mathrm{Tr}\left[\rho_{B}^{\kappa }⊗|\kappa \rangle {\langle \kappa |}_{A_{e}}V\left(|\Psi \rangle \langle \Psi |\right)\right]\leq \sum_{\kappa =0}^{D-1}\frac{1}{d}\langle \kappa |\rho_{A_{e}}|\kappa \rangle =\frac{1}{d} \end{array}$$

since the largest overlap of a *d*-dimensional maximally entangled state with a product state is $\frac{1}{d}$.

We now show that the upper bound in Equation (31) is tight for when using the maximally entangled state |Ψ〉 as the resource state. In this case, observe that

(33) $$\rho_{B}^{\kappa }={\mathrm{Tr}}_{A_{e}}\left[\left(I_{B}⊗|\kappa \rangle \langle \kappa |\right)V\left(|\Psi \rangle \langle \Psi |\right)\right]=\frac{1}{d}V^{†}{\left(|\kappa \rangle \langle \kappa |\right)}^{*}.$$

Substituting this into Equation (30) gives

(34) $$\begin{array}{cc} {F_{\mathrm{cl}}}^{\left(\mathrm{EDC}\right)}= & \left(1-q\right)\left(1-p\right)+\left(1-q\right)\frac{p}{d}\mathrm{Tr}\left[(\sum_{\kappa =0}^{D-1}V^{†}{\left(|\kappa \rangle \langle \kappa |\right)}^{*}⊗V^{†}\left(|\kappa \rangle \langle \kappa |\right)|\Psi \rangle \langle \Psi |)\right]+\frac{q}{dD}\\ = & \left(1-q\right)\left[\left(1-p\right)+\frac{p}{d}\right]+\frac{q}{dD}. \end{array}$$

Note that SDC is the special case of EDC where d=D. A depiction of communication fidelity to message size trade-off can be seen in Figure 1. □

### Noisy Singlet Resource

To show a more general result, we will examine the Classical Correlation Fidelity of EDC and SDC in the case where Alice and Bob’s shared resource is a noisy singlet of the form

(35) $$\rho^{\left(d\right)}=\theta |\Psi \rangle \langle \Psi |+\left(1-\theta \right)\frac{I⊗I}{d^{2}}\;\mathrm{such}\;\mathrm{that}\;0<\theta \leq 1.$$

Observe that

(36) $$\rho_{B}^{\kappa }={\mathrm{Tr}}_{A_{e}}\left[\left(I_{B}⊗|\kappa \rangle \langle \kappa |\right)V\left(|\Psi \rangle \langle \Psi |\right)\right]=\frac{1}{d}V^{†}{\left(|\kappa \rangle \langle \kappa |\right)}^{*}.$$

Substituting this into Equation (30) gives

(37) $$\begin{array}{cc} {F_{\mathrm{cl}}}^{\left(\mathrm{EDC}\right)}= & \left(1-q\right)\left(1-p\right)+\left(1-q\right)\frac{p}{d}\mathrm{Tr}\left[(\sum_{\kappa =0}^{D-1}V^{†}{\left(|\kappa \rangle \langle \kappa |\right)}^{*}⊗V^{†}\left(|\kappa \rangle \langle \kappa |\right)|\Psi \rangle \langle \Psi |)\right]+\frac{q}{dD}\\ = & \left(1-q\right)\theta \left[\left(1-p\right)+p\frac{1}{d}\right]+\left(1-q\right)\left(1-\theta \right)\frac{1}{d^{2}}+\frac{q}{dD}. \end{array}$$

We can therefore say, the Classical Correlation Fidelity when using a one parameter noisy singlet state is

(38) $${F_{\mathrm{cl}}}^{\left(\mathrm{EDC}\right)}=\left(1-q\right)\left(\left(1-p\right)+\theta \frac{p}{d}+\left(1-\theta \right)\frac{p}{d^{2}}\right)+\frac{q}{dD}.$$

Furthermore, since SDC is a special case of EDC where d=D we can generalize to say

(39) $${F_{\mathrm{cl}}}^{\left(\mathrm{SDC}\right)}=\left(1-q\right)\left(\left(1-p\right)+\theta \frac{p}{D}+\left(1-\theta \right)\frac{p}{D^{2}}\right)+\frac{q}{D^{2}}.$$

This more general result demonstrates that the advantage identified in Proposition 1 are not exclusive to the ideal resource case but generalized to additional non-ideal resource states. This extension of our result strengthens our claim that EDC provides a general communication advantage to SDC. Note that, as shown in Appendix A, this represents the optimal fidelity achievable by the noisy singlet resource since the utilized POVM is optimized.

## 6. Noisy Local Operations: Hardware Implementation

We now evaluate EDC in the case where Alice and Bob are connected by a noiseless quantum channel but have limited local capabilities and are susceptible to local errors. To accomplish this analysis, we implement a modified version of EDC on current-day quantum hardware. We find that EDC serves as a more efficient alternative to SDC in terms of transmission success probability when implemented using non-ideal noisy local systems.

### 6.1. Decoding Gate Volume and Noise Accumulation

We implemented an adapted Embedded Dense Coding (EDC) scheme on IBM’s Heron processor to explore its advantages in noisy settings. Throughout this section, we describe the protocol in terms of logical qudits. On current superconducting hardware, these logical qudits are realized using collections of physical qubits; the details of this encoding are discussed in Section 6.2. This section evaluates the relationship between the resulting gate volume and the accumulation of noise during execution. Experimentally, it is hard to isolate the impact from a specific type of quantum noise, such as dephasing. Results in this section will therefore include the accumulation of all types of noise affecting modern quantum hardware.

Practically, the primary source of noise for protocols on current hardware is the use of two-qubit gates, typically with a probability of error on the order of $10^{-3}$ per control Z gate (basis two-qubit gate) on IBM Eagle and Heron processors [24]. In dense coding protocols, these are used for the preparation of the initial entangled state and Alice’s decoding of the received message. Therefore, the quantity of these two-qubit operations scales linearly with the dimension of the entanglement dimension *d*, contributing directly to the accumulated physical error.

Embedded Dense Coding reduces this overhead by using an embedded resource state, which lowers the entanglement dimension *d* required for transmitting the same effective message dimension. Because the total accumulated error grows approximately linearly with the number of noisy two-qudit gates, a reduction in decoding depth yields a proportional reduction in the internal hardware noise.

Thus, EDC’s reduced decoding Gate Volume translates to a lower expected error contribution from native device noise without requiring any change to the physical hardware. This expectation is supported by the results of our implementation on IBM’s Heron processor through ${\mathrm{IBM}}_{\mathrm{Torino}}$ in Figure 2.

### 6.2. Implementation and Results

Due to modern hardware constraints, we utilize multiple physical qubits to encode logical qudits rather than employing native higher-dimensional physical qudits. This means that, to simulate a *D*-dimensional qudit, we use $\log_{2}\left(D\right)$ qubit channels, which have a total of *D* possible configurations. For example, a ququart is encoded using two qubit channels as such:

(40) $$\begin{array}{ccccccc} |0\rangle \mapsto |00\rangle & \; & |1\rangle \mapsto |01\rangle & \; & |2\rangle \mapsto |10\rangle & \; & |3\rangle \mapsto |11\rangle \end{array}$$

Further adaptations followed from qubit encoding, such as how Pauli gates and higher-dimensional embeddings are applied, which can be found in Appendix B.

Using this implementation, we conducted a comparison of success probabilities when transmitting the same number of classical bits of information via EDC protocols with various entanglement dimensions *d*. We performed six independent trials of 10,000 shots per trial, which yielded an average variance between trials of approximately 1.2%, confirming the stability of the simulation. The results of these tests can be seen in Figure 2, where each trajectory reflects a distinct embedding strategy and illustrates the associated success probability as the number of encoded messages increases. Figure 2 highlights the inherent trade-off between success rate and entanglement dimension introduced by the embedding process. Specifically, the observed divergence in success probabilities provides insight into the performance constraints encountered when attempting to encode messages in extended Hilbert space architectures.

Although these results are indicative of EDC advantages, due to the difficulty of implementing true qudit quantum codes, we can not make concrete assertions about EDC’s performance if implemented via true qudit quantum codes. These results have been included due to their suggestive nature but require additional exploration in the future as quantum hardware advances.

## 7. Conclusions

Embedded dense coding is a generalization of deterministic dense coding, which embeds one subsystem into a higher-dimensional Hilbert space, increasing the classical capacity of an entanglement resource. In this work, we compare the performance of EDC with SDC and find that EDC offers fidelity advantages and additional flexibility when compared to SDC.

To assess performance in realistic conditions, we first evaluated EDC under a dephrasure channel acting on the transmitted system while assuming ideal local operations. To enable a meaningful comparison with SDC, we fixed the classical communication rate per shared entangled resource and compared the resulting one-shot Classical Correlation Fidelity. Under this criterion, we show that when the entanglement dimension is strictly smaller than the channel dimension, EDC achieves a lower transmission error than SDC in the presence of dephasing and loss. This result establishes a clear noise–rate trade-off: decreasing the entanglement-to-channel dimension ratio reduces the number of distinguishable messages but improves robustness to dephasing.

We additionally implemented an adapted EDC scheme on IBM’s Heron processors to examine its behavior under contemporary hardware noise. In this setting, the communication channel itself is effectively noiseless, and imperfections arise primarily from gate errors and circuit depth. Consequently, the experimental study does not directly simulate the dephrasure model analyzed theoretically. Instead, it evaluates how the structural differences between EDC and SDC influence accumulated hardware error. We observe that embeddings requiring reduced decoding gate volume yield higher empirical success probabilities compared to conventional dense coding circuits of comparable dimension. Although constrained by the lack of native qudit support, these results demonstrate that the architectural features of EDC can translate into measurable performance gains on current qubit-based platforms.

Embedded Dense Coding provides a dimension-expanding reformulation of dense coding that preserves optimal noiseless performance while introducing a tunable trade-off between communication rate and noise robustness. Analytically, it reduces transmission error relative to SDC under dephrasure noise at a fixed rate, and experimentally, it benefits from reduced decoding complexity under hardware noise. Together, these results clarify the operational role of entanglement dimension in dense coding protocols and highlight embedding as a meaningful design parameter for near-term quantum communication settings.

## Figures and Tables

**Figure A1:**
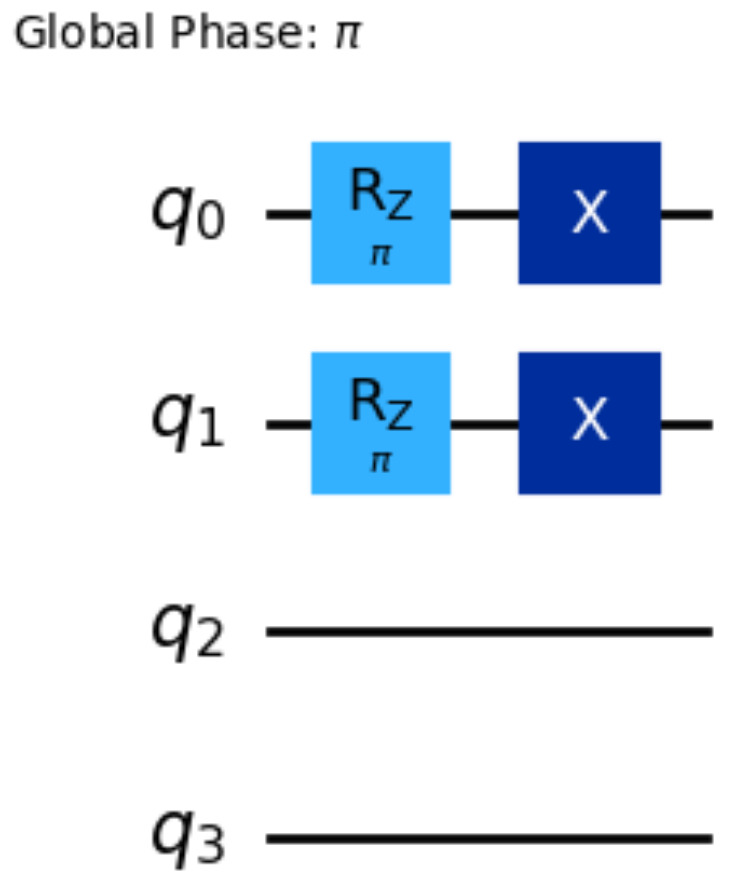
A $Z_{4}^{3}$ (**left**) and $X_{4}^{3}$ (**right**) gate used in a d=D=4 EDC protocol. Both gates are expressed in terms of the native gates on the IBM Heron processor. As is seen, when d=D=4, a maximum of only four native gates is used for the encoding of the message.

**Figure A2:**
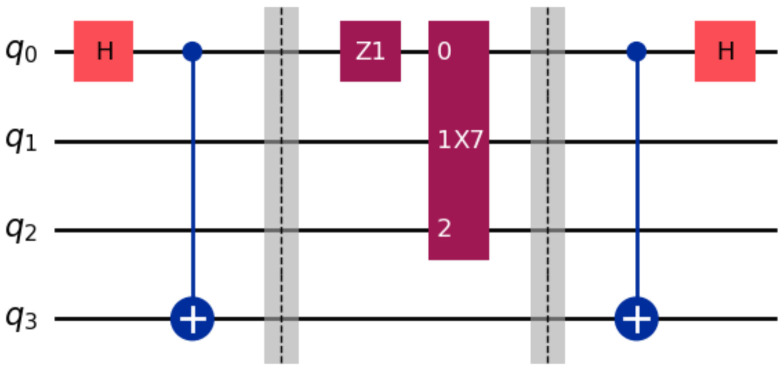
Example of Embedded Dense Coding implementation on IBM Heron processor through ${\mathrm{IMB}}_{\mathrm{Torino}}$ using starting dimension d=2 and embedding dimension D=8, resulting in a total of four classical bits of information being transmitted.

**Figure A3:**
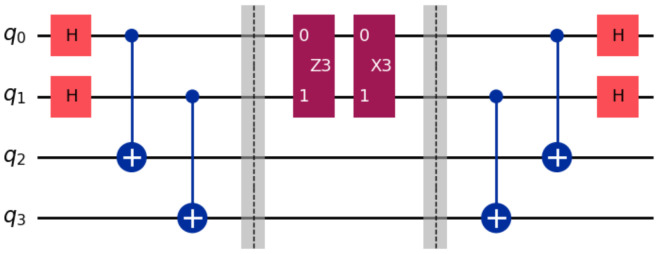
Example of conventional Dense Coding implementation on IBM Heron processor through ${\mathrm{IMB}}_{\mathrm{Torino}}$ using starting dimension d=4, resulting in a total of four classical bits of information being transmitted. Note, this represents the special case of EDC where d=D=4.

## Data Availability

The original contributions presented in this study are included in the article. Further inquiries can be directed to the corresponding author.

## Abbreviations

The following abbreviations are used in this manuscript:

EDC	Embedded Dense Coding
SDC	Standard Dense Coding
FEF	Fully Entangled Fraction
CCF	Classical Correlation Fidelity
MES	Maximally Entangled State

## Appendix A. Optimality Constraints

We will show that the physically motivated POVM utilized in EDC is optimal for any dephrasure channel $N_{p,q}$ if Alice and Bob share an entangled resource of the form of the canonical one-parameter noisy singlet:

(A1) $$\rho^{\left(d\right)}=\theta |\Psi \rangle \langle \Psi |+\left(1-\theta \right)\frac{I⊗I}{d^{2}}\;\mathrm{such}\;\mathrm{that}\;0<\theta \leq 1.$$

**Proof.** As shown by [25], a necessary and sufficient condition for some POVM ${\left\{\Pi^{\mu_{i}}\right\}}_{i=0}^{dD-1}$ to be optimal in the state discrimination problem ${\left\{\varrho_{\mu_{i}}^{\prime \prime }\right\}}_{i=0}^{dD-1}$ is for

(A2) $$\sum_{i}p_{i}{\hat{\varrho }}^{\mu_{i}}\Pi^{\mu_{i}}-p_{j}{\hat{\varrho }}^{\mu_{j}}\geq 0,\;\forall j.$$

This requirement is equivalent to the condition

(A3) $$\langle \psi |\frac{1}{dD}\sum_{i}{\hat{\varrho }}^{\mu_{i}}\Pi^{\mu_{i}}|\psi \rangle \geq \frac{1}{dD}\langle \psi |{\hat{\varrho }}^{\mu_{j}}|\psi \rangle \;\forall |\psi \rangle ,\;\forall j.$$

Note that, without loss of generality, since any POVM that detects erasure will perform equally in the case of erasure, we can assume q=0. Starting with the LHS, we can use our result from Equation (28) to say,

(A4) $$\begin{array}{cc} \mathrm{LHS} & =\langle \psi |\frac{1}{dD}\sum_{i}{\hat{\varrho }}^{\mu_{i}}\Pi^{\mu_{i}}|\psi \rangle \\ & =\frac{1}{dD}\langle \psi |\sum_{i=0}^{dD-1}\left(\mathrm{id}⊗E^{\mu_{i}}\right)\left(\left(1-p\right)V\left(\rho^{\left(d\right)}\right)+p\left(\sum_{\kappa =0}^{D-1}\varrho_{B}^{\kappa }⊗|\kappa_{A_{e}}\rangle \langle \kappa_{A_{e}}|\right)\right)\left(\mathrm{id}⊗E^{\mu_{i}}\right)V\left(\Psi \right)|\psi \rangle \\ & =\frac{1}{dD}\langle \psi |\sum_{i=0}^{dD-1}\left(\mathrm{id}⊗E^{\mu_{i}}\right)[(\left(1-p\right)V\left(\rho^{\left(d\right)}\right)\\ & +p\left(\sum_{\kappa =0}^{D-1}\varrho_{B}^{\kappa }⊗|\kappa_{A_{e}}\rangle \langle \kappa_{A_{e}}|\right))\left(I⊗{\left(X_{A_{e}}^{j}{\hat{Z}}_{A_{e}}^{k}\right)}^{†}\left(X_{A_{e}}^{j}{\hat{Z}}_{A_{e}}^{k}\right)\right)V\left(\Psi \right)]|\psi \rangle \end{array}$$

Note that $I⊗{\left(X_{A_{e}}^{j}{\hat{Z}}_{A_{e}}^{k}\right)}^{†}\left(X_{A_{e}}^{j}{\hat{Z}}_{A_{e}}^{k}\right)=VV^{†}$ is the projection operator onto the image Im(V), and since V(Ψ) is in the image of *V*, we can say,

(A5) $$\begin{array}{cc} \mathrm{LHS}= & \frac{1}{dD}\langle \psi |\sum_{i=0}^{dD-1}\left(\mathrm{id}⊗E^{\mu_{i}}\right)\left[\left(\left(1-p\right)V\left(\rho^{\left(d\right)}\right)+p\left(\sum_{\kappa =0}^{D-1}\varrho_{B}^{\kappa }⊗|\kappa_{A_{e}}\rangle \langle \kappa_{A_{e}}|\right)\right)V\left(\Psi \right)\right]|\psi \rangle \\ = & \frac{1}{dD}\langle \psi |\sum_{i=0}^{dD-1}\left(\mathrm{id}⊗E^{\mu_{i}}\right)\left[\left(\left(1-p\right)V\left(\rho^{\left(d\right)}\right)V\left(\Psi \right)+p\left(\sum_{\kappa =0}^{D-1}\varrho_{B}^{\kappa }⊗|\kappa_{A_{e}}\rangle \langle \kappa_{A_{e}}|\right)V\left(\Psi \right)\right)\right]|\psi \rangle \\ = & \frac{1}{dD}\langle \psi |\sum_{i=0}^{dD-1}\left(\mathrm{id}⊗E^{\mu_{i}}\right)\left[\left(\left(1-p\right)V\left(\theta \Psi +\frac{\left(1-\theta \right)}{d^{2}}\Psi \right)+pV\left(\frac{\theta }{d}\Psi +\frac{\left(1-\theta \right)}{d^{2}}\Psi \right)\right)\right]|\psi \rangle \\ = & \frac{1}{dD}\langle \psi |\sum_{i=0}^{dD-1}\left(\mathrm{id}⊗E^{\mu_{i}}\right)\left[V\left(\theta \left(1-p+\frac{p}{d}\right)\Psi +\frac{\left(1-\theta \right)}{d^{2}}\Psi \right)\right]|\psi \rangle \end{array}$$

Note that, ${\left\{\left(\mathrm{id}⊗E^{\mu_{i}}\right)V\left(\Psi \right)\right\}}_{i=1}^{dD-1}$ forms a complete basis in our Hilbert space. Thus,

(A6) $$\mathrm{LHS}=\frac{1}{dD}\left(\theta -\theta p+p\frac{\theta }{d}+\frac{\left(1-\theta \right)}{d^{2}}\right).$$

We can now evaluate the RHS,

(A7) $$\begin{array}{cc} \mathrm{RHS}= & \frac{1}{dD}\langle \psi |{\hat{\varrho }}^{\mu_{j}}|\psi \rangle \\ = & \frac{1}{dD}\langle \psi |\left(\mathrm{id}⊗E^{\mu_{j}}\right)\left(\left(1-p\right)V\left(\rho^{\left(d\right)}\right)+p\left(\sum_{\kappa =0}^{D-1}\varrho_{B}^{\kappa }⊗|\kappa_{A_{e}}\rangle \langle \kappa_{A_{e}}|\right)\right)|\psi \rangle . \end{array}$$

We will now find the maximum value of the RHS. Note that our encoding operator, as an isometry, should not affect the maximum overlap; thus, we can say,

(A8) $$\begin{array}{cc} \max_{|\psi \rangle }\mathrm{RHS}= & \frac{1}{dD}\max_{|\psi \rangle }\langle \psi |\left(\left(1-p\right)V\left(\rho^{\left(d\right)}\right)+p\left(\sum_{\kappa =0}^{D-1}\varrho_{B}^{\kappa }⊗|\kappa_{A_{e}}\rangle \langle \kappa_{A_{e}}|\right)\right)|\psi \rangle . \end{array}$$

This is maximized when |ψ〉〈ψ|∈ImV(·), thus we can utilize our pre-embedding dimension since the isometry preserves the inner product,

(A9) $$\begin{array}{cc} \max_{|\psi \rangle }\mathrm{RHS}= & \frac{1}{dD}\max_{|\psi \rangle }\langle \psi |\left(\left(1-p\right)\rho +p(\sum_{\kappa =0}^{d-1}\rho_{B}^{\kappa }⊗|\kappa_{A}\rangle \langle \kappa_{A}|)\right)|\psi \rangle \\ = & \frac{1}{dD}\max_{|\psi \rangle }[\left(1-p\right)\left(\theta \langle \psi |\Psi |\psi \rangle +\frac{\left(1-\theta \right)}{d^{2}}\right)\\ + & p(\theta \langle \psi |\sum_{\kappa =0}^{d-1}\Psi_{B}^{\kappa }⊗|\kappa_{A}\rangle \langle \kappa_{A}||\psi \rangle +\frac{\left(1-\theta \right)}{d^{2}})]\\ = & \frac{1}{dD}\max_{|\psi \rangle }\left[\left(1-p\right)\left(\theta \langle \psi |\Psi |\psi \rangle \right)+p\left(\theta \langle \psi |\sum_{\kappa =0}^{d-1}\Psi_{B}^{\kappa }⊗|\kappa_{A}\rangle \langle \kappa_{A}||\psi \rangle \right)+\frac{\left(1-\theta \right)}{d^{2}}\right]. \end{array}$$

We can see that this is maximized when |ψ〉=|Ψ〉, and, thus,

(A10) $$\max_{|\psi \rangle }\mathrm{RHS}=\frac{1}{dD}\left(\left(1-p\right)\theta +p\frac{\theta }{d}+\frac{\left(1-\theta \right)}{d^{2}}\right).$$

From Equations (A6) and (A10), we can therefore see that LHS =max RHS meaning

(A11) $$\sum_{i}p_{i}{\hat{\varrho }}^{\mu_{i}}\Pi^{\mu_{i}}-p_{j}{\hat{\varrho }}^{\mu_{j}}\geq 0,\;\forall j.$$

is true and our POVM is optimized for a noisy singlet regardless of dephasure. □

## Appendix B. Implementation on Quantum Hardware

Due to the limitation that qubit encoding must be used, we performed adjustments to the rest of the protocol to ensure it still behaved as expected, the first of these being the way embedding is handled. In the typical EDC Protocol, higher-dimensional embedding is only applied after the *Z* gates are applied and involves the introduction of another, higher-dimensional quantum channel. In our implementation, Alice’s qudit begins by possessing the necessary quantum channels to simulate a *D*-dimensional qudit; they just remain unused until the *X* gates are applied, conserving the notion that embedding only happens after entanglement and the *Z* operation. In other words, qubit channels corresponding to the extra D−d dimensions added after embedding remain untouched until the *X* gate is applied.

Another adaptation was applied to the way generalized Pauli *Z* and Pauli *X* gates are applied. Custom unitary Qiskit gates using the matrix definitions shown in Equations (4) and (5) involve the use of many native gates and, in turn, make the protocol very noisy. Instead, the *d* different *Z* gates we define are simply the *d* possible combinations of applying qubit *Z* gates to each qubit of our encoding. For example, for d=4, $Z_{4}^{0}$ is defined as not applying any *Z* gate to any qubit of the encoding, and $Z_{4}^{3}$ is defined as applying a *Z* gate to both qubits of the encoding. For the $X_{D}$ gates, the same applies only with *D* possibilities instead of *d*, usually involving more qubit channels due to the encoding (unless d=D). An example of a $Z_{4}^{3}$ and $X_{4}^{3}$ gate is shown in Figure A1.

This way of expressing gates is only possible when both *d* and *D* are powers of two. Otherwise, custom unitary gates must be used to ensure that no qudit reaches a forbidden state (such as 〈11| in the case of D=3 qutrits). As these gates present much worse performance, we restrict ourselves to only cases where *d* and *D* are powers of two.

Restricting *D* and *d* allows for Bob’s decoding of the message to be very simple. Reversing the order of the gates used for entanglement is enough to decode the message such that none of Bob’s particles are found in a superposition. Bob can therefore measure both particles and always get the same outcome (assuming no noise). A few examples of EDC protocols with different values of d,D can be seen in Figure A2 and Figure A3.
